# Recipient Hyperbilirubinemia May Reduce Ischemia-Reperfusion Injury but Fails to Improve Outcome in Clinical Liver Transplantation

**DOI:** 10.1155/2016/6964856

**Published:** 2016-05-22

**Authors:** Mihai Oltean, Christian Barrenäs, Paulo Ney Martins, Gustaf Herlenius, Bengt Gustafsson, Styrbjörn Friman, William Bennet

**Affiliations:** ^1^The Transplant Institute, Sahlgrenska University Hospital, 413 45 Gothenburg, Sweden; ^2^Sahlgrenska Academy, The University of Gothenburg, Gothenburg, Sweden; ^3^Department of Surgery, Transplant Division, University of Massachusetts, Worcester, MA 01655, USA

## Abstract

*Background.* Exogenous bilirubin may reduce experimental ischemia-reperfusion injury (IRI) due to its antioxidant properties. We studied if early graft exposure to high bilirubin levels in the recipient affects the early IRI and outcomes after liver transplantation (LTx).* Methods.* In 427 LTx patients, the AUROC curve based on bilirubin and AST at day 1 identified a cutoff of 2.04 mg/dL for the recipient pretransplant bilirubin. Recipients were grouped as having low (group L, *n* = 152) or high (group H, *n* = 275) bilirubin. Both groups had similar donor-related variables (age, preservation time, donor BMI > 28, and donor risk index (DRI)).* Results.* Alanine (ALT) and aspartate (AST) aminotransferase levels were higher in group L at day 1; ALT levels remained higher at day 2 in group L. LTx from high risk donors (DRI > 2) revealed a trend towards lower transaminases during the first two days after transplantation in group H. One month and 1-year patient survival were similar in groups L and H. High preoperative bilirubin did not affect the risk for early graft dysfunction (EGD), death, or graft loss during the first year after transplantation nor the incidence of acute rejection. LTx using donors with DRI > 2 resulted in similar rates of EGD in both groups.* Conclusion.* Increased bilirubin appears to reduce the early IRI after LTx yet this improvement was insufficient to improve the clinical outcome.

## 1. Introduction

Ischemia-reperfusion injury (IRI) of the liver graft reemerges as a major issue as the pressure of ever increasing waiting lists requires the use of extended criteria donor livers with steatosis, older, and non-heart-beating donors. These grafts are more susceptible to IRI with up to 10% of the procured livers not being transplanted due to the risk of primary graft nonfunction or dysfunction and ischemic type cholangiopathy [1]. Several protective strategies aimed at inducing biological protective mechanisms in the graft or recipient before the injurious chain of events in the graft occurs (i.e., ischemic or pharmacologic preconditioning) have been explored both in clinical and in experimental settings [2–5]. Although promising, many of these approaches rely on complex protocols or toxic chemicals or are technically demanding and expensive (machine perfusion). This often renders them unpractical and limits their clinical applicability.

Bilirubin is a byproduct of heme catabolism occurring through the heme oxygenase-1 pathway. Bilirubin has been shown to have potent antioxidant properties [6, 7] and exogenous administration of bilirubin in several models of isolated organ perfusion has been shown to have a protective effect against IRI when administered before or during the ischemic event [8, 9]. Experimental evidence indicates that even a brief contact with bilirubin is sufficient to confer protection against IRI [8]. Furthermore, a clinical report suggests a protective effect of increased endogenous bilirubin against development of late graft failure after kidney transplantation [10]. Although the protective mechanisms were unidentified, the protective effect could be due to the reduction of the oxidative stress associated with IRI resulting in reduced reperfusion injury.

Hyperbilirubinemia is regarded as a negative prognostic factor in chronic liver disease and is a component of the Model for End-stage Liver Disease (MELD) reference system, widely used for allocation of organs for liver transplantation [11, 12]. However, hyperbilirubinemia may be of benefit in the very early period after transplantation by mitigating the oxidative stress upon reperfusion. Therefore, the aim of this study was to investigate if early graft exposure to high bilirubin levels in the recipient would have any impact on the early reperfusion injury and outcomes after liver transplantation.

## 2. Patients and Methods

### 2.1. Study Population

We studied retrospectively all adult patients who underwent transplantation with a liver from a brain-dead donor, liver as a single, ABO-compatible, whole organ at the Transplant Institute at Sahlgrenska University Hospital between January 2003 and December 2010. We excluded from the analysis pediatric patients, patients undergoing simultaneous transplantation of other organs, living donor liver transplants, split liver transplants, portocaval hemitransposition, or early technical failures/intraoperative deaths.

### 2.2. Donor Information

Information on the organ donors was obtained from the organ report form and Scandiatransplant, the common transplant registry of Sweden, Norway, Finland, Denmark, and Iceland. Donor data included information on donor gender, weight, length, cause of death, preservation solution, and cold ischemia time (CIT). The donor risk index (DRI) was computed according to an acknowledged equation, using a calculator available online [13, 14]. Concerning the “organ location” parameter in the formula, the organs procured in Gothenburg and in hospitals within 60 km were considered as “local,” organs procured at other donor hospitals from our procurement area (Western, Southern, and Northern Sweden) were considered as “regional,” and organs shared from other centers within the Scandiatransplant cooperation were inputted as “national.”

### 2.3. Liver Transplantation

We retrospectively reviewed patient files and retrieved information on patient demographics, underlying disease, MELD, and surgical technique. We also recorded the length of stay on the intensive care unit and rejection during the first month after transplantation as well as one-month and one-year graft and patient survival. The MELD score refers to the MELD based on laboratory values obtained shortly prior to transplantation.

The patients were divided into two groups according to their pretransplant bilirubin levels following a post hoc analysis using a receiver operating characteristic- (ROC-) curve. Reperfusion injury was estimated according to the level of aspartate aminotransferase (AST) and alanine aminotransferase (ALT) on posttransplantation days 1, 2, and 7. Early graft dysfunction (EGD) was defined according to Olthoff et al. with standard criteria as maximum alanine transferase (ALT) or aspartate transferase (AST) levels > 2000 U/L during the first week, international normalized ratio (INR) ≥ 1.7, or bilirubin > 200 micromol/L at the end of the first postoperative week [15].

Immunosuppression consisted in tacrolimus, mycophenolate mofetil, and steroids until January 2010 and a steroid-free protocol thereafter.

### 2.4. Statistical Analysis

Results are presented as mean ± standard deviation (unless otherwise specified). A receiver operating characteristic (ROC) curve was constructed by plotting the sensitivity versus 1 − specificity using the preoperative bilirubin and ALT at day 1 to calculate the area under the ROC curve and to identify a cutoff value of bilirubin.

Following the Kolmogorov-Smirnov test for normality distribution, differences between groups were assessed with Student's *t*-test for normally distributed variables or the nonparametric *U* test (Mann-Whitney) was used for nonnormal distributed variables. Categorical variables (cause of death, gender, acute rejection, and retransplantation rate) were assessed using Fisher's exact test. Differences were considered statistically significant when the *p* value was <0.05. The statistical software used was GraphPad Prism for Windows Version 5.00 (GraphPad Software, Inc., San Diego, CA, USA).

## 3. Results

Five hundred eighty-six liver transplants were performed at the Sahlgrenska University Hospital in Gothenburg between January 1st 2003 and December 31st, 2010. One hundred fifty-nine liver transplants were excluded based on the exclusion criteria. A post hoc analysis of the remaining 427 patients using the ROC curve identified a recipient pretransplant bilirubin concentration of 2.04 mg/dL (35 micromol/L) as the cutoff value. Accordingly, the patients (and corresponding donors) were grouped in a low bilirubin group, group L (bilirubin < 2.04 mg/dL, *n* = 152), and a high bilirubin group, group H (bilirubin ≥ 2.04 mg/dL, *n* = 275).

Donor and graft characteristics did not differ significantly between groups in terms of age, cold ischemia time, donor BMI > 28, DRI, or preservation solution.

There was no significant difference between the recipients in the two groups before transplant except for MELD score and pretransplant diagnosis. Donor- and recipient-related variables are summarized in Table 1.

### 3.1. Posttransplant Transaminase Levels in the First Week as a Surrogate Marker of Ischemia-Reperfusion Injury

**Figure 1:**
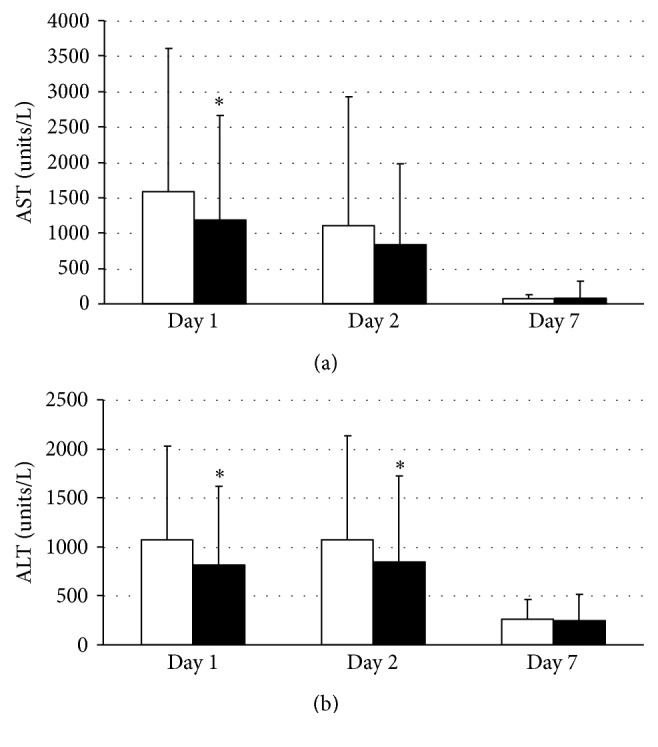
Serum transaminases in the first week after transplantation in patients with low (group L, white bar) and high pretransplant bilirubin (group H, black bar); (a) aspartate aminotransferase (AST); (b) alanine aminotransferase (ALT); ^*∗*^ *p* < 0.05.

Patients in group L have had significantly higher ALT and AST at day 1 than patients in group H. ALT levels remained higher at day 2 in group L while AST showed only a trend towards higher values (*p* = 0.08). One week after transplantation plasma transaminases reached similar values in the two groups (Figure 1).

The subgroup analysis of the liver transplants from donors with DRI > 2 revealed a trend towards lower transaminases during the first two days after transplantation in recipients with high preoperative bilirubin. One-month and 1-year graft survival were similar in groups L and H (93.4% versus 94.8, *p* = n.s., and,88.1 versus 87.9%, *p* = n.s.), respectively. High preoperative bilirubin did not have any impact on the risk of death or graft loss during the first year after transplantation.

The incidence of early graft dysfunction (EGD) as defined by Olthoff et al. did not differ significantly between the two groups (65.7% in group L versus 60% in group H, *p* = n.s.). Increased preoperative bilirubin did not significantly alter the risk for EGD (RR 0.912; OR 0.78 *p* = 0.25).

The rate of early retransplantation (within one month) was similar in both groups (8/152 in group L versus 9/275 in group H, *p* = n.s.). Primary graft nonfunction requiring retransplantation did not differ significantly between groups (5/152 in group L versus 4/275 in group H, *p* = n.s.). High preoperative bilirubin did not impact the incidence or the risk for acute rejection within the first month after transplantation (RR 1.166: OR 1.24 *p* = 0.37).

Recipients in group H had a longer stay on the intensive care unit compared with group L (6.7 ± 3 versus 3.5 ± 2 days, *p* < 0.05).

A subgroup analysis of the liver transplants from donors with DRI > 2 indicated a similar incidence of EGD irrespective of the pretransplant bilirubin level in the recipient. However, the rates of retransplantation at one month and at one year were higher in group L. Patient survival at one month and that one year after transplantation were similar in the two subgroups (Table 2).

## 4. Discussion

The results of the study suggest that high preoperative bilirubin levels in liver graft recipients may decrease the early biochemical signs of reperfusion injury. However, this improvement did not suffice to improve the outcome.

Ischemia-reperfusion injury with early graft dysfunction (EGD) or the primary graft nonfunction (PNF) is a serious concern following liver transplantation. PNF is life threatening and requires urgent retransplantation and thus utilizing two livers for the same patient also impacts other patients on the liver waiting list.

Experimental studies indicate that exposure to byproducts of the heme oxygenase-1 pathway such as carbon monoxide, biliverdin, or bilirubin or the upregulation of heme oxygenase- (HO-) 1 expression may mitigate ischemia-reperfusion injury in various organs [16–18]. Exogenous bilirubin has potent antioxidant and protective properties in IRI models even at milimolar concentrations. This interesting hypothesis may have a straightforward biochemical explanation, since bilirubin may undergo oxidation to biliverdin at reperfusion, when intense oxidative stress ensues [6]. Thus, increased amounts of cytoprotective biliverdin may be rapidly generated without increasing the amount of noxious ferrous iron.

Donor-recipient selection and matching is an important topic when establishing optimal allocation strategies [19, 20]. Patients in group H had significantly higher MELD score and one may have expected grafts of better quality for these sicker patients. However, the grafts in the two groups had comparable donor-related variables (age, DRI, preservation solution, cold ischemia time, or BMI > 28 as a surrogate for steatosis). Thus, organ quality as the cause behind the observed differences in the IRI markers can be excluded. However, despite biochemical signs of a milder reperfusion injury, patients with higher bilirubin (and subsequently higher MELD) had a more complicated postoperative course, reflected by the longer median ICU stay and the early mortality. This finding is neither surprising nor new and indicates that whatever supposed cytoprotection bilirubin may entail it is insufficiently potent to outweigh the challenges of early posttransplant period [21].

Experimental HO-1 upregulation has demonstrated a marked antioxidant effect, allowing a more efficient degradation of the toxic, prooxidant heme molecule [22]. Moreover, experimental administration of either carbon monoxide or biliverdin to either organ donors or recipients greatly reduced reperfusion injury as well as the occurrence of acute and chronic rejection and it has been suggested that all these compounds may have intrinsic cytoprotective, antiapoptotic, and immunomodulatory effects [23–25]. It seems that even a brief contact with bilirubin improves preservation injury, through mechanisms mimicking HO-1 preconditioning [8].

The induction of HO-1 demonstrated immunomodulatory effect in various transplantation models and reduced the frequency of acute rejection in different transplant models [26–28]. Our study did not find any significant difference between the two groups in incidence of acute rejection during the first month implying that bilirubin and/or any putative mechanisms did not change (either increase or decrease) the immunogenicity of the livers in the two groups. Alternatively, the potent immunosuppressive regimen used throughout the study overshadowed any theoretical benefits of bilirubin or its byproducts and compensated for any presumed immunomodulatory effect in recipients with low bilirubin.

An earlier study investigated the protective role of increased preoperative bilirubin against IRI and found no relationship between the preoperative bilirubin and IRI (transaminase leak) [29]. However, the study of Manzinate et al. has several drawbacks that may have led to some incorrect conclusions. The study pooled peak AST values at different postoperative days during the first five days instead of studying them at specific time points. In addition, the study did not attempt any grouping according to bilirubin levels nor did it analyze important confounding factors such as donor age, BMI, and cold ischemia time. The controversy is further fueled by a recent study conducted in living donor liver transplantation which confirmed the protective effects of increased bilirubin against ischemia-reperfusion injury but did not find clinical outcome [30].

The current findings partly confirm the existing evidence on a beneficial effect of bilirubin against IR injury, yet the effect is mild and has an unclear clinical significance. This cytoprotective effect remained low when livers with high DRI were analyzed separately. Most evidence on bilirubin-induced protection against IRI comes from experimental studies, utilizing young, healthy animals. The experimental setting offers the possibility of a tightly controlled timing and dose of the preconditioning agent, whereas our study resembles more a per- and postconditioning protocol. Moreover, the livers in the clinical studies endure the immunological storm secondary to the brain death and are submitted to an additional injury disregarded by virtually any animal study.

A drawback of the current study is pooling the different types of bilirubin (direct and indirect) instead of studying them separately. This originates in the retrospective nature of the study and the existing clinical routines that do not differentiate between the different fractions (total bilirubin). It is generally acknowledged that unconjugated bilirubin has the highest lipophilic and antioxidant properties, though all bilirubin types may have antioxidant properties [7, 31]. Although the amount of unconjugated bilirubin is dependent on many factors and it is low in the healthy individual (<20%), the increase in total bilirubin during various liver diseases regularly leads to high levels of unconjugated, indirect bilirubin. Thus, one study found an initial difference of 10 mg/dL between the total and conjugated bilirubin in patients with liver failure undergoing albumin dialysis [32]. Therefore, we can expect that high amounts of indirect bilirubin were present in group H at the time of transplantation.

Another limitation of the study is the lack of histopathology analysis to score graft steatosis. At our center, we do not perform routinely preimplantation biopsies and only in selected cases is a biopsy taken to assess the degree of steatosis. Nonetheless, we used donor BMI as a surrogate marker of the liver steatosis as BMI over 28 has been suggested to be predictive of moderate or severe liver steatosis [33]. Since graft biopsies are performed at the end of the procedure after variable periods of reperfusion and IRI may be in different stages of development we did not use graft biopsies to score IRI. Previous reports confirmed the ability of transaminases to reflect the degree of IRI in clinical liver transplantation; hence, we used transaminases alone to assess the hepatocellular injury after reperfusion [34]. We limited our recipient analysis to the MELD score and did not include other possible recipient risk factors for IRI (e.g., pressors, acidosis, and infections). However, by excluding portocaval hemitransposition or split livers from the analysis, we avoided potential significant source of transaminase leak or postoperative comorbidities in our series. We interpret IRI as the major cause of the transaminase elevation following liver transplantation in our study.

## 5. Conclusions

Increased bilirubin appears to reduce the early biochemical signs of reperfusion injury after liver transplantation. However, this improvement is insufficient to impact decisively the early and late clinical outcome.

## Figures and Tables

**Table 1:** Donor and recipient characteristics.

	Group low (*n* = 152)	Group high (*n* = 275)	Significance
*Donor*			
Age (SD)	52.3 ± 15	51.5 ± 16	n.s.
Gender (M/F, %)	55.3/44.7	51.1/48.9	n.s.
BMI > 28 (%)	15.2	12	n.s.
Preservation solution			
(UW/Custodiol/other, %)	48/52/0	60/48.4/0.6	
DRI (mean, SD)	1.67 ± 0.37	1.7 ± 0.39	n.s.
Cold ischemia time (min, mean ± SD)	504 ± 185	519 ± 157	n.s.
Cause of death			
Trauma (%)	13.16	10.55	
Anoxia (%)	13.16	8.73	
Cerebrovascular accident (%)	65.79	60	
Other (%)	7.89	20.73	
*Recipient*			
Age	53.5 ± 10	50.7 ± 12.4	n.s.
MELD (mean)	9	20.5	0.001
Diagnosis			
Hepatocellular cancer, other tumors (%)	29.6	7.2	0.001
Postviral cirrhosis (%)	37.5	23.6	0.01
Sclerosing cholangitis (%)	19.1	21.8	0.05
Primary biliary cirrhosis (%)	2	10	0.01
Laennec cirrhosis (%)	15.8	17	n.s.
Cryptogenic cirrhosis (%)	6.6	8.3	n.s.
Acute hepatic failure (%)	0.6	7.3	0.01
Retransplantation (%)	6.6	12	n.s.
Other (%)	19.8	11	
Surgical technique			
Standard OLT	5.3	2.9	n.s.
OLT with venovenous bypass	19.7	26.9	n.s.
Piggyback technique	75	70.2	n.s.

**Table 2:** An overview of liver biochemistry (transaminase leak as surrogate marker for the reperfusion injury) as well as the early and late transplant outcome in recipients receiving livers from high risk donors (DRI ≥ 2, *n* = 83).

	Low bilirubin (<2.04 mg/L) (*n* = 25)	High bilirubin (>2.04 mg/L) (*n* = 58)	*p* value
*AST day 1* (±SD)	2358 ± 3882	1194 ± 1411	0.02
*AST day 2* (±SD)	1894 ± 3411	852 ± 1176	0.05
*AST day 7* (±SD)	70 ± 58	70 ± 41	0.99
*ALT day 1* (±SD)	1205 ± 1117	894 ± 823	0.09
*ALT day 2* (±SD)	1370 ± 1411	911 ± 882	0.15
*ALT day 7* (±SD)	258 ± 235	211 ± 117	0.33
*EGD* (*n*, %)	11 (44%)	24 (41%)	1
*Re-Tx within 1 month* (*n*, %)	5 (20%)	1 (1.8%)	0.01
*Re-tx within 1 year* (*n*, %)	7 (28%)	4 (7.5%)	0.01
*Death within 1 month* (*n*, %)	0	1 (1.8%)	1
*Death within 1 year* (*n*, %)	1 (4%)	4 (6.8%)	1
